# Blood donation landscape in Bulgaria: data analytics insights from 2024

**DOI:** 10.1098/rsfs.2025.0092

**Published:** 2026-08-28

**Authors:** Ivo Umlenski, Vassia Atanassova, Peter Vassilev, Tania Pencheva, Nikolay Nikolov, Nikolay Andreev, Simeon Ribagin, Vasilina Vasileva, Nora Angelova, Angel Dimitriev, Krassimir Atanassov, Diana Petkova

**Affiliations:** ^1^ Institute of Biophysics and Biomedical Engineering Bulgarian Academy of Sciences, Sofia, Bulgaria; ^2^ Department of Transfusion Haematology - Pernik, Pernik, Bulgaria; ^3^ SMDL ImmunoChem, Sofia, Bulgaria; ^4^ Regional Centre of Transfusion Haematology - Pleven, Pleven, Bulgaria; ^5^ Faculty of Mathematics and Informatics, Sofia University Saint Kliment Ohridski, Sofia, Bulgaria

**Keywords:** transfusion haematology, blood group distributions, ABO system, blood donation, voluntary blood donation, donor recruitment strategies

## Abstract

The paper presents data and analysis of the blood group distributions of the Bulgarian population for the year 2024, retrieved from the national information system serving the centres and departments of transfusion haematology. Several significant indicators are considered in both national and regional aspects and in both absolute numbers and percentages. The study is particularly focused on tracing the ABO system blood type frequencies, Rh(D) antigen (rhesus factor) frequencies and certain demographic indicators of blood donation activity, including blood donor's sex, age, location and type of donation (voluntary or family/replacement). The atomic approach towards data analysis, adopted as early as the research planning phase, has given vast opportunities for data stratification in various ways and for discovering patterns, regional specifics and even data anomalies. The presented data and conclusions can serve in planning the operation and resources of the centres and departments of transfusion haematology on national and regional levels, for information backup of the blood donation campaigns and for raising the society's awareness and sensibility towards the needs and benefits of voluntary blood donation as the safest and most sustainable source of blood supply.

This article is part of the theme issue ‘Data driven modelling for living systems’.

## 1. Introduction

Efficient blood supply management depends on a comprehensive and continuously updated understanding of population-level immunohaematological characteristics and patterns of blood donation activity. In contemporary transfusion medicine, maintaining accurate national and regional estimates of ABO blood group frequencies and Rh(D) antigen distribution is essential for forecasting demand, anticipating shortages and optimizing inventory management. Reliable estimates of the prevalence of Rh(D)-negative and less common blood groups are particularly important for ensuring the availability of compatible components for patients with specific transfusion requirements. These immunohaematological parameters should be intersected with detailed demographic information on donor populations—including sex, age, geographic location and donation type (voluntary versus family/replacement)—as demographic and behavioural factors can significantly influence donation rates and the availability of specific blood components.

Despite the importance of these indicators, systematic and data-driven analyses of blood group distributions and blood donation practices in Bulgaria remain limited. However, such information is needed to support targeted recruitment and retention strategies and public-awareness campaigns encouraging regular voluntary donation, particularly among under-represented donor groups and individuals with rare or clinically important blood types. This need is well exhibited in Bulgaria, along with other countries in Eastern Europe, where for historical reasons the blood donation landscape in past decades has substantially changed for the worse. In the late socialism (pre-1989), blood donation was massively promoted and organized through workplaces, the army, universities and trade unions and linked to civic duty campaigns. The subsequent transition period (1990s–early 2000s) was marked by a significant drop in voluntary donation and increased reliance on family/replacement donors, which was a result from economic crisis and institutional restructuring, emigration and demographic changes, decline of the public trust in institutions and collapse of workplace-based mass campaigns. From the 2000s onward, more reliable statistics and published figures give a clearer picture [1], yet voluntary donation is still dominated by family/replacement donation, shortages remain common and slight increases in the numbers per capita are more likely to result from declining population instead of increased blood donation culture.

Recent advances in data analytics and data-driven decision-making approaches have provided deeper understanding and powerful tools for identifying temporal and spatial patterns and for quantifying associations between donor characteristics and donation behaviours. A granular approach to the analysis of donation dynamics can support improved blood donor recruitment strategies, policy development and operational decision-making on national and regional levels. The present study applies statistical and data-visualization techniques to analyse the 2024 dataset of Bulgarian blood donors, capturing blood group distributions, Rh(D) antigen frequencies and demographic indicators on both regional and national levels. By examining these indicators individually and in combinations, the study aims to characterize the current landscape of blood donation activity, identify regional and demographic disparities and provide an updated overview of transfusion haematology indicators at a national level, as well as ideas for communication and recruitment strategies addressing specific target groups of the population.

In this context, the findings provide an updated evidence base that may assist in optimizing blood-collection planning and donor-management strategies in Bulgaria, contributing to improved transfusion services, more efficient logistics and inventory management of whole blood and blood products, risks assessment and more informed decision-making within the national transfusion haematology and healthcare system.

## 2. Material and methods

The paper presents the 2024 data from the centres of transfusion haematology (CTHs) and departments of transfusion haematology (DTHs) in Bulgaria, a total number of 29 divisions, grouped into five regional centres, and discusses these data in several significant aspects, on both national and regional scales, as well as in both absolute values and percentages.

The network of CTHs and DTHs in Bulgaria (figure 1) is organized as follows: one National Centre of Transfusion Haematology (NCTH) in the capital city of Sofia, within which operate eight subordinate districts’ DTHs, covering western Bulgaria, and four Regional Centres of Transfusion Haematology (RCTHs), each comprising its respective subordinate DTHs: RCTH Plovdiv with four subordinate DTHs (south-central Bulgaria), RCTH Stara Zagora with three subordinate DTHs (south-eastern Bulgaria), RCTH Pleven with five subordinate DTHs (north-central Bulgaria) and RCTH Varna with four subordinate DTHs (north-eastern Bulgaria). All departments in the network correspond to the administrative regional centres of the country, with the sole exception of the town of Lom, which was the traditional transfusion haematology unit for Montana District until 2024, when a new department in the town of Montana itself was constituted. This organizational structure will be used for the presentation of all data in the article, unless specified otherwise.

**Figure 1.**
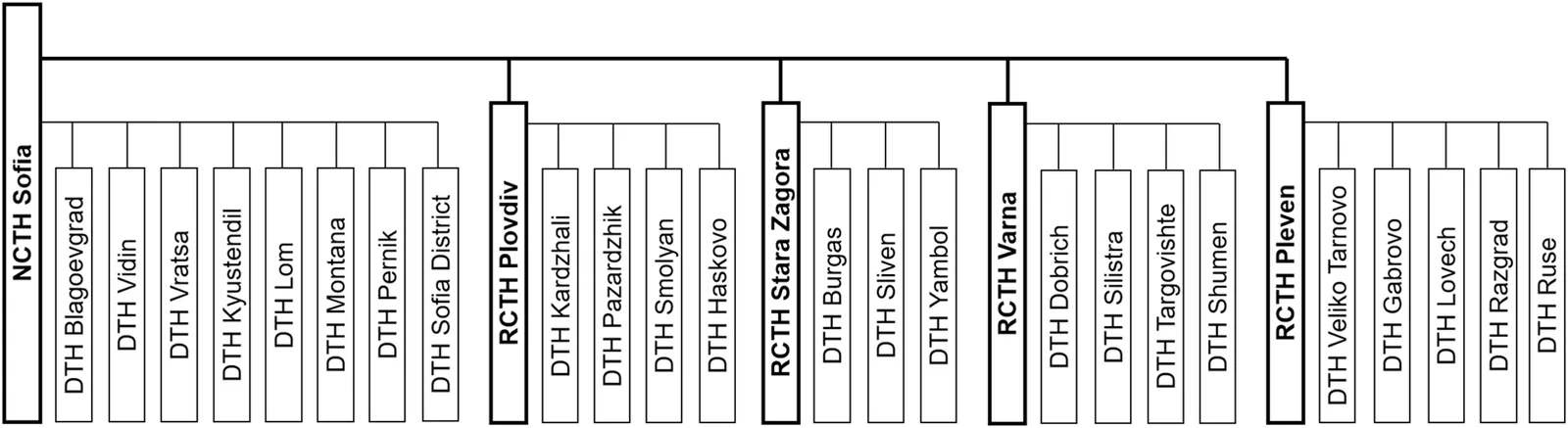
Structure of the Bulgarian national system of transfusion haematology: one NCTH, four RCTHs and 24 subordinate district departments.

The present study encompasses a selection of 172 570 registered blood donations in Bulgaria in 2024, of which 129 689 were from male donors and 42 881 were from female donors. All analyses were conducted exclusively on pre-anonymized data. The dataset of 178 514 donations reported by the NCTH for 2024 [2] underwent preliminary data analysis and cleaning. During this process, 5890 records corresponding to apheresis donors and remunerated donors were removed. In addition, a small number of records (54) were excluded owing to missing (non-imputable) or incorrectly entered (irreparable) values for some of the major traced indicators (donor's blood group, Rh(D), sex and/or type of donation).

There has been strict compliance with all requirements set out in Ordinance No. 18 of 2004 of the Bulgarian Ministry of Health regarding blood samples, test reagents and test erythrocytes used for determining blood group characteristics. Determination of ABO blood group and Rh(D) antigen is included in the immunohaematological diagnostics. Data on other investigated erythrocyte antigens outside the ABO and Rh(D) systems are not considered in this study.

The atomic approach to data analysis, incorporated as early as the planning stage of the research, offers significant opportunities for stratifying the data in diverse ways, identifying patterns and regional specificities and even detecting data errors and anomalies. Since such an atomic approach is largely absent from the relatively few Bulgarian studies published on the subject in the past, direct comparison between current and earlier data is not always possible; nevertheless, where such comparison can be made, it constitutes a valuable contribution to both the science and practice of transfusion haematology.

## 3. Results and discussion

### 3.1. ABO and Rh(D) blood group distributions

Maintaining accurate and up-to-date information on ABO and Rh(D) blood group distributions within a given country's population is important for a wide range of reasons and has practical implications for blood-service management. Blood group compatibility is crucial for ensuring safe transfusion practices and avoiding adverse immune reactions in ABO-compatible non-identical transfusions [3]. Availability of national and regional blood group distribution data helps forecasting and managing blood and blood product supplies, thereby facilitating more effectively the processes of planning and organization of voluntary blood donation campaigns to secure adequate quantities of the most in-demand groups for transfusion—both according to their frequency in the population and in view of the universal emergency transfusion group O(–) [4,5]. Closely related is the factor of preparedness at both national and local levels to respond to disasters and emergencies, when blood and blood product reserves may sharply decrease within short periods of time, necessitating careful planning under conditions of limited or depleted stocks. In addition, studies have shown that individuals with certain blood groups may be associated with increased or decreased susceptibility to specific diseases [6−9].

Data derived from the NCTH information system, based on registered blood donations in Bulgaria in 2024, yield the following nationwide averages (table 1, figure 2a), summarized according to the ABO system: blood group O: 33.84%, blood group A: 41.56%, blood group B: 16.65% and blood group AB: 7.95%. When the Rh(D) antigen (Rhesus factor) is included, the blood type frequencies are as follows: O(–) 4.47%, O(+) 29.37%, A(–) 5.21%, A(+) 36.35%, B(–) 2.15%, B(+) 14.50%, AB(–) 1.00% and AB(+) 6.95%. The national mean ratio between Rh(–) and Rh(+) antigens across all ABO groups is 12.83% to 87.17%, and the group-specific ratios are additionally calculated and presented in figure 2b.

**Table 1.** Blood type distributions in Bulgaria, 2024: country average values, per sex and altogether, compared with the regional minimal and maximal values by blood type. All numerical values are in %.

Blood type	Males average	Females average	Country average	Minimal value	DTH with minimal value	Maximal value	DTH with maximal value
**O(–)**	4.32	4.94	4.47	3.55	DTH Pazardzhik	8.50	DTH Smolyan
**O(+)**	29.37	29.36	29.37	24.13	DTH Kyustendil	34.62	DTH Smolyan
**A(–)**	5.01	5.81	5.21	4.56	DTH Burgas	6.63	DTH Montana
**A(+)**	36.61	35.55	36.35	32.13	DTH Sliven	39.07	DTH Ruse
**B(–)**	2.08	2.35	2.15	1.70	DTH Haskovo	3.19	DTH Gabrovo
**B(+)**	14.64	14.09	14.50	12.30	DTH Gabrovo	17.45	DTH Sliven
**AB(–)**	0.96	1.13	1.00	0.68	DTH Burgas	2.07	DTH Silistra
**AB(+)**	7.00	6.77	6.95	2.88	DTH Smolyan	8.76	DTH Kyustendil

**Figure 2.**
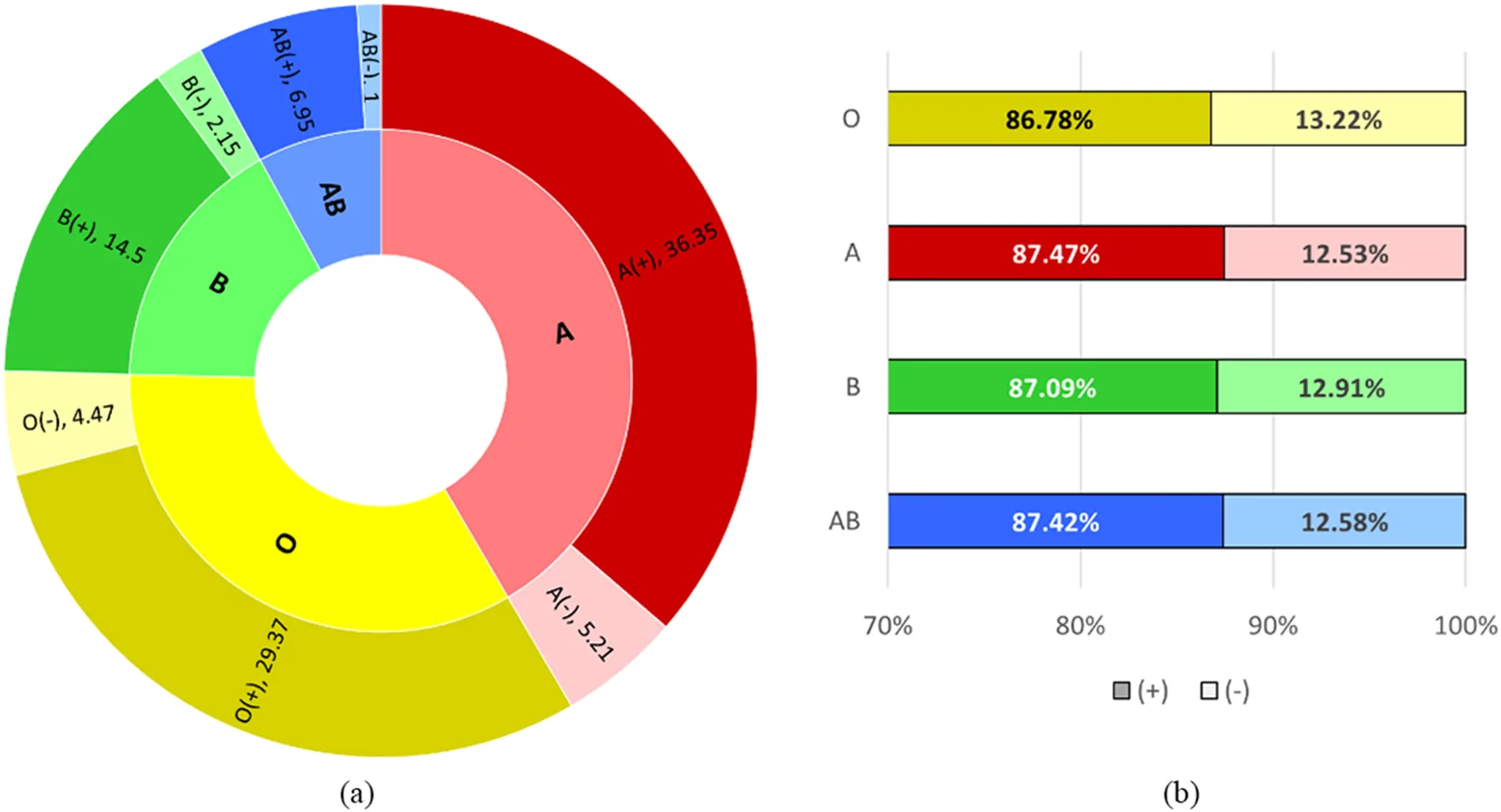
Blood type distributions in Bulgaria, 2024, in %: (a) all ABO blood types; (b) Rh(D) frequencies.

A detailed presentation of the blood group distributions by administrative districts, based on data from registered blood donations in 2024, is provided in table 2, which also includes information on the number of individual blood donors per district.^1^ In addition to the district-level percentage distribution of the eight blood types defined by the ABO system and the Rh(D) antigen, the table also presents aggregated data for the four ABO blood groups and the ratios of Rh(–) and Rh(+) frequencies. The nationwide averages (last row of the table) are subsequently illustrated in figure 3 by means of a Sankey diagram, which, to the best of current knowledge, represents a novel yet highly informative method for presenting immunohaematological data.

**Table 2.** District-level blood type distributions in Bulgaria in 2024, with aggregated data columns for ABO blood groups and Rh(D) antigen frequencies, in %.

CTH / DTH of transfusion haematology	Total blood donations	O(‒)	O(+)	A(‒)	A(+)	B(‒)	B(+)	AB(‒)	AB(+)	O	A	B	AB	Rh(‒)	Rh(+)
**NCTH SOFIA**	**41 869**	4.36	28.86	5.10	36.98	1.92	14.65	0.96	7.16	33.22	42.08	16.58	8.12	12.34	87.66
DTH Blagoevgrad	7475	4.55	29.47	5.46	36.21	2.13	14.72	0.95	6.52	34.02	41.67	16.84	7.46	13.08	86.92
DTH Vidin	1263	3.80	30.11	6.58	34.79	2.06	13.55	1.66	7.45	33.91	41.36	15.61	9.11	14.10	85.90
DTH Vratsa	2055	6.28	26.37	6.57	34.65	2.48	15.91	1.61	6.13	32.65	41.22	18.39	7.74	16.93	83.07
DTH Kyustendil	1589	4.66	24.13	5.42	38.82	1.83	15.31	1.07	8.76	28.80	44.23	17.14	9.83	12.98	87.02
DTH Lom	1810	4.86	29.56	5.30	34.59	2.76	14.64	1.05	7.24	34.42	39.89	17.40	8.29	13.98	86.02
DTH Montana	984	5.10	29.49	6.63	32.86	3.06	14.90	1.22	6.73	34.59	39.49	17.96	7.96	16.02	83.98
DTH Pernik	1781	4.16	28.87	5.35	35.28	2.25	15.14	0.90	8.05	33.03	40.63	17.39	8.95	12.66	87.34
DTH Sofia District	4254	4.35	28.37	5.41	37.15	2.02	14.22	0.97	7.51	32.72	42.56	16.24	8.47	12.76	87.24
**RCTH PLOVDIV**	**26 679**	4.08	32.37	4.77	35.54	2.69	12.71	1.46	6.37	34.18	41.69	16.50	7.63	12.59	87.41
DTH Kardzhali	2746	3.55	28.84	5.14	37.08	1.98	14.85	0.75	7.81	36.45	40.31	15.40	7.83	13.00	87.00
DTH Pazardzhik	5353	8.50	34.62	5.73	32.42	2.23	12.88	0.73	2.88	32.39	42.22	16.83	8.56	11.41	88.59
DTH Smolyan	2603	4.68	32.50	5.52	35.34	1.70	13.24	0.98	6.05	43.12	38.15	15.12	3.62	17.19	82.81
DTH Haskovo	3588	4.21	29.71	5.27	35.01	2.42	15.01	1.04	7.33	37.18	40.86	14.94	7.02	12.88	87.12
**RCTH VARNA**	**13 691**	4.00	29.61	4.80	35.59	1.94	15.58	1.74	6.74	33.92	40.28	17.43	8.37	12.93	87.07
DTH Dobrich	2524	5.11	27.57	5.67	35.20	2.78	14.97	2.07	6.63	33.61	40.39	17.52	8.48	12.49	87.51
DTH Silistra	1977	4.69	28.63	4.79	33.42	2.80	15.84	1.53	8.30	32.68	40.87	17.75	8.70	15.63	84.37
DTH Targovishte	1969	4.03	29.82	5.30	34.52	2.47	15.45	1.30	7.11	33.32	38.21	18.64	9.83	13.81	86.19
DTH Shumen	3153	4.83	30.51	5.02	35.89	2.43	14.07	0.79	6.45	33.85	39.82	17.93	8.41	13.10	86.90
**RCTH S. ZAGORA**	**7702**	3.85	29.71	4.56	37.90	2.01	14.19	0.68	7.12	35.34	40.91	16.50	7.24	13.07	86.93
DTH Burgas	9180	4.54	30.27	5.03	32.13	2.19	17.45	1.03	7.35	33.55	42.46	16.19	7.79	11.08	88.92
DTH Sliven	3875	3.67	29.38	5.59	35.86	3.01	14.88	1.21	6.41	34.81	37.16	19.64	8.39	12.80	87.20
DTH Yambol	2562	4.04	28.40	5.69	37.81	2.38	13.54	0.96	7.17	33.05	41.45	17.89	7.62	13.48	86.52
**RCTH PLEVEN**	**8730**	4.43	30.22	5.41	37.06	1.87	12.91	1.01	7.08	32.44	43.50	15.92	8.13	13.08	86.92
DTH Veliko Tarnovo	3363	5.46	28.15	5.05	37.32	3.19	12.30	1.16	7.37	34.65	42.47	14.78	8.09	12.73	87.27
DTH Gabrovo	1723	5.52	29.95	5.22	36.31	2.12	14.07	0.91	5.90	33.60	42.37	15.50	8.53	14.86	85.14
DTH Lovech	1322	5.30	27.86	5.14	34.75	1.91	16.63	1.27	7.15	35.48	41.53	16.19	6.81	13.77	86.23
DTH Razgrad	1888	4.99	28.08	5.29	39.07	1.99	13.23	0.90	6.45	33.16	39.88	18.54	8.42	13.61	86.39
DTH Ruse	4916	4.36	28.86	5.10	36.98	1.92	14.65	0.96	7.16	33.07	44.36	15.22	7.35	13.17	86.83
**Total/Average**	**172 570**	**4.47**	**29.37**	**5.21**	**36.35**	**2.15**	**14.50**	**1.00**	**6.95**	**33.84**	**41.56**	**16.65**	**7.95**	**12.83**	**87.17**

**Figure 3.**
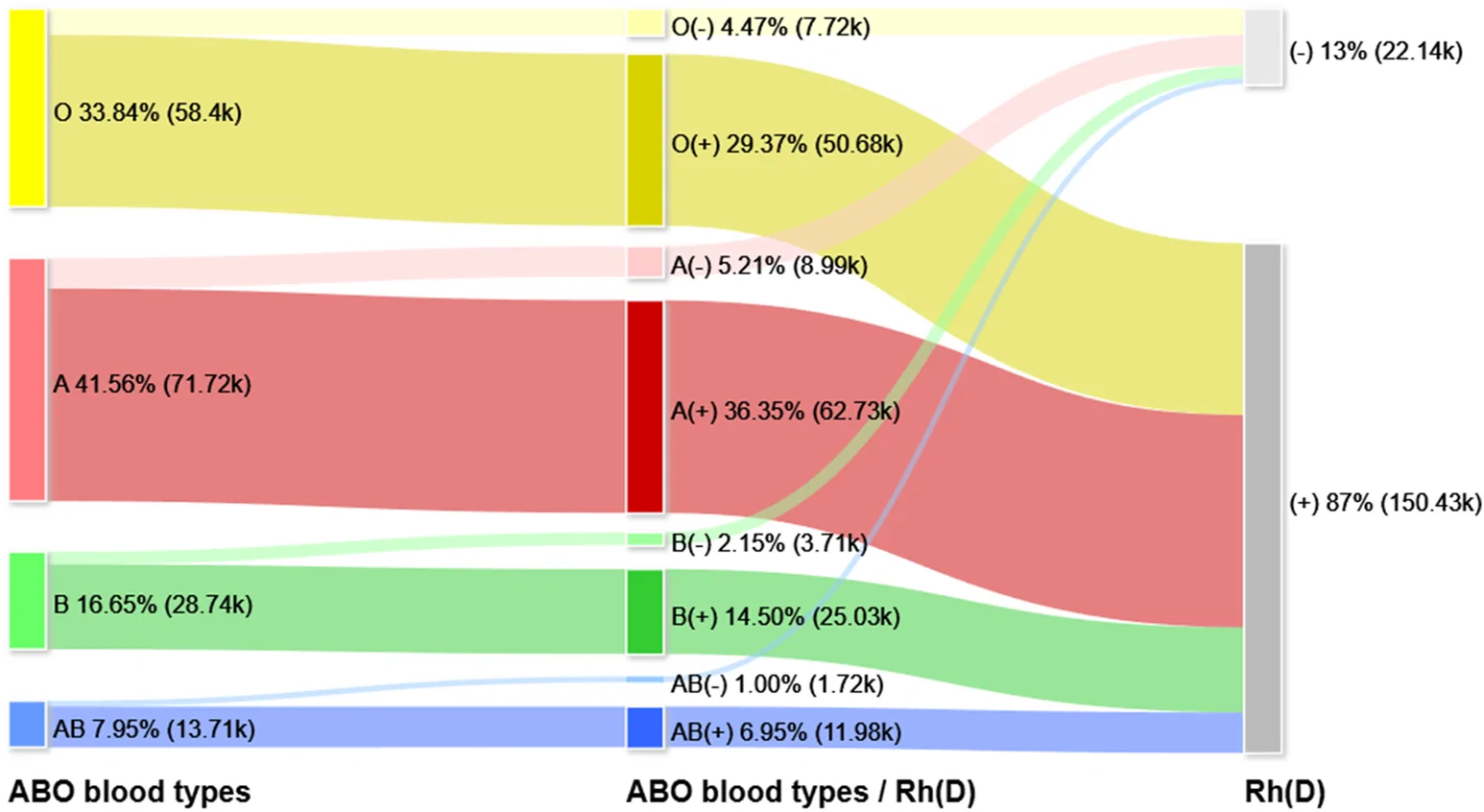
Sankey diagram of the nation-level average blood type distributions in Bulgaria in 2024.

Discussion of the presented results is most naturally done in the light of comparison with preceding literature data. The blood group distribution data established in 2024 are illustrated and compared in figure 4, with data published by other authors in 1957 [10], 1977 [11] and 2012 [12], and with publications by some of the authors of the present study, based on datasets spanning the periods 2015–2020 [13], 2015–2021 [14] and 2023 [15], respectively.

**Figure 4.**
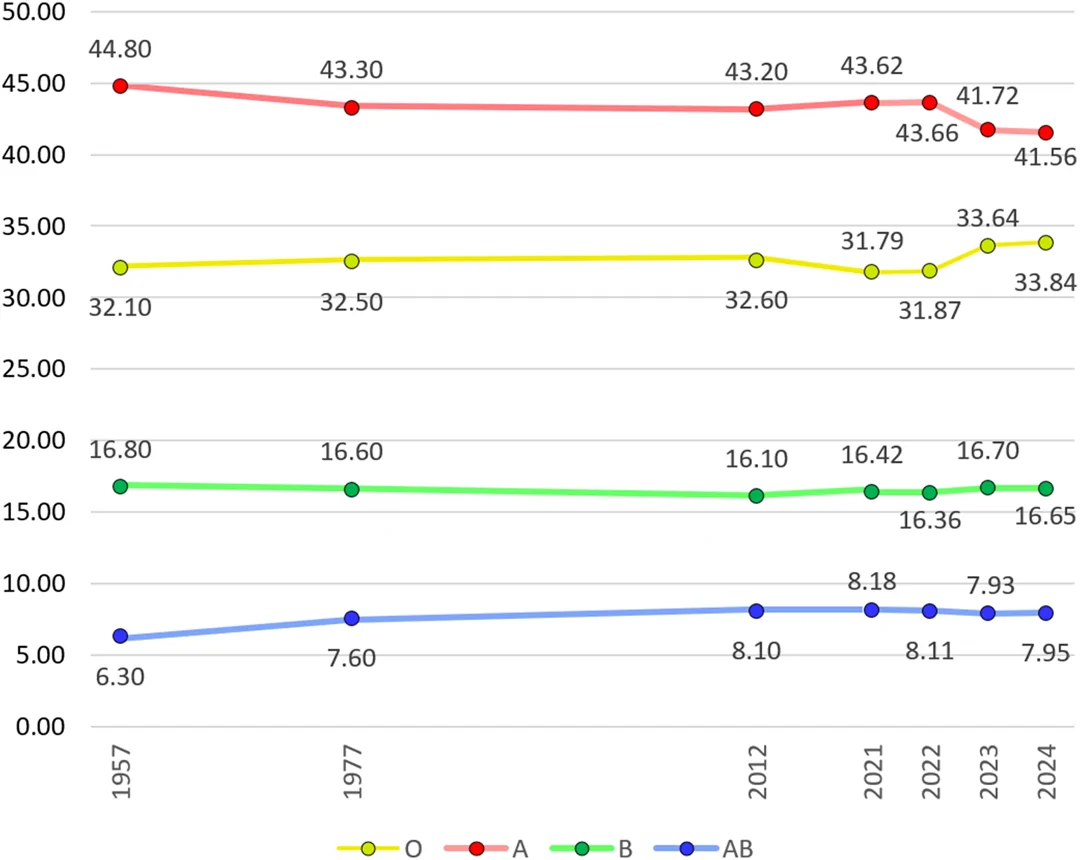
Comparison of the present data (2024) for the blood types distribution (in %) in Bulgaria with data from previously published literature sources in 1957 [10], 1977 [11], 2012 [12], 2021 [13], 2022 [14] and 2023 [15].

A noticeable change in the data for blood groups O and A beginning in 2023 is highlighted in comparison with the data from the relatively long preceding period, and in 2024, these two values remain essentially the same. Basically, most of the explanation is in the methodology of collecting the data: in the present paper and in [15], the data are from the blood donations recorded by the NCTH on a national level, whereas in [13] and [14], data were based on patients of the University Hospital “Saint Anna” in Sofia. For comparison, source [12] reports data based on both blood donors and patients of the Military Medical Academy, Sofia, while sources [10,11] do not specify the methods of data collection and the sizes of the investigated human cohorts, and thus in many aspects do not allow long-term comparability. All these observations and considerations underscore the need for a detailed nationwide retrospective study that could put these findings and trends into a practical context.

### 3.2. Blood donation activity by district/location

A major problem faced nowadays by the Bulgarian blood transfusion system, and therefore by the national healthcare system, is the population's relatively low engagement in blood donation and limited awareness of the society's needs of blood and blood products, as well as of the personal health benefits from regular voluntary donation. As a result, blood has become a scarce resource, making the systematic monitoring of donors’ activity patterns and demographic profiles an essential component of effective transfusion management, i.e. identifying local disparities and ensuring the timely availability of blood products where they are most needed.

Statistics on blood donations per 1000 inhabitants are maintained internationally by the World Health Organization (WHO), and according to a 2011 report, estimates for Europe ranged from 6 to 67.6 donations per 1000 inhabitants, with the highest donation activity recorded in Denmark [16]. According to the WHO's reports covering the period 2002–2012, blood donations in Bulgaria increased from 18.22 to 22.83 per 1000 inhabitants. This upward trend has reflected both the gradual growth in the absolute number of donations (142 951 in 2002 [17, p. 86], 152 813 in 2004 [17, p. 28], 153 337 in 2005 [17, p. 86], 153 644 in 2007 [18, pp. 8–9], 162 658 in 2011 [19, p. 14] and 166 324 in 2012 [20, p. 71]), as well as the parallel process of population decline owing to ageing and emigration. According to the WHO, for a country to be self-sufficient in blood supply, it is estimated to maintain a minimum of 20 donors per 1000 inhabitants [16].

Analysis of the 2023 data from the NCTH information system [15] revealed that this indicator had risen to 25.90 donations per 1000 inhabitants, based on 166 962 donations and a total national population of 6 445 481 [21]. For RCTH Pleven solely, even more detailed data for the period 2017–2022 are available in [22] and [23].

Using data from the information system of the Bulgarian NCTH, an average of 26.81 blood donations per 1000 inhabitants was calculated for 2024, derived from 172 570 registered blood donors and a total population of 6 437 360 for that year, according to the National Statistical Institute. Compared with WHO recommendations, this figure indicates that Bulgaria's blood donation rate is below the levels typically required for self-sufficiency and well under the benchmarks observed in countries with well-developed voluntary donation cultures. Such a rate suggests structural vulnerabilities in the blood supply, increased dependence on repeat donors, limited capacity to manage fluctuations in demand or unexpected shortages, especially considering demographic pressures and increasing clinical demand.

This nation-level average can be additionally ‘zoomed in’ and analysed on a regional level (table 2). Being the state capital, Sofia City (hosting the NCTH Sofia) expectedly contributes the greatest number of blood donations, accounting for about one quarter (24.25%) of all donations in the country, while the NCTH Sofia together with its eight subordinate DTHs (collectively covering western Bulgaria) contributes more than one third (36.54%) of all donations nationwide. RCTH Plovdiv, along with its four subordinate DTHs (comprising south-central Bulgaria), contributes another 23.73% of all blood donations, while RCTH Varna (north-eastern Bulgaria), RCTH Pleven (north-central Bulgaria) and RCTH Stara Zagora (south-eastern Bulgaria) with their respective subordinate DTHs complete the statistics with their 13.51%, 13.51% and 12.71% of the country's blood donations, respectively. In most general figures, the total blood donations per district are visualized spatially on the choropleth map of Bulgaria (figure 5).

**Figure 5.**
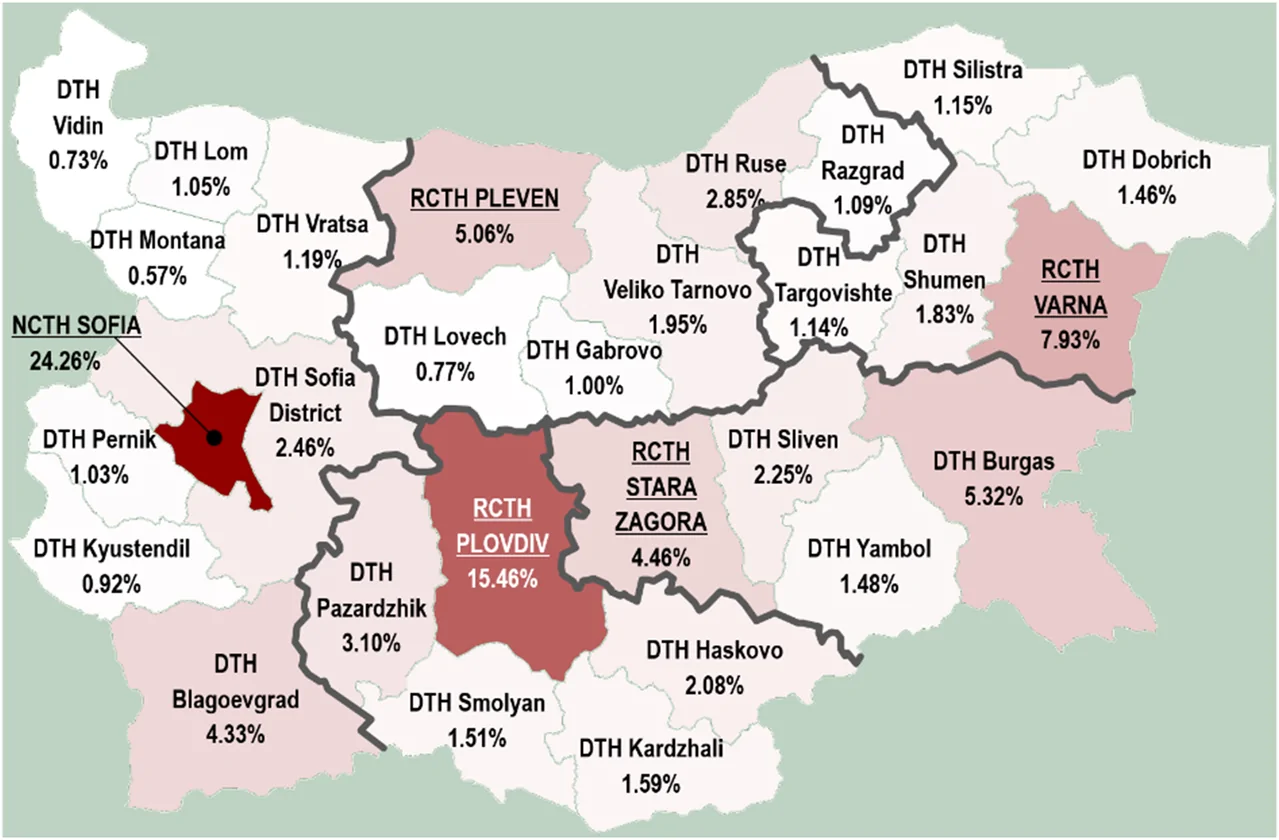
Choropleth map of Bulgaria regarding the total number of blood donations, in %

In terms of basic descriptive statistics, the dataset shows the characteristics of a moderately heavy-tailed distribution, showing a mean of 0.0357, a standard deviation of 0.0495 and a Gini coefficient of 0.5350, with one significant outlier (Sofia, *p* < 0.05), confirming a long tail of substantially small values and a high level of concentration of a few large values, contributing disproportionately to the total.

The picture of blood donation activity changes when the absolute number of donations by districts is weighted against the population size of the respective districts (according to data from the National Statistical Institute for 2024 [21]), i.e. expressed as the number of donations per 1000 inhabitants as presented in table 3. In order to highlight the differences between the two approaches, the data are shown in two ways: separately sorted in descending order with respect to the absolute numbers of blood donation acts and in per-capita numbers, i.e. numbers of blood donations per 1000 inhabitants.

**Table 3.** District-level numbers of blood donations: in absolute terms and in 1000 inhabitants

Sorted by indicator (1) ‘Blood donations in absolute values’			Sorted by indicator (2) ‘Blood donations per 1000 inhabitants’		
**CTH**/DTH	**(1)**	**(2)**	**CTH**/DTH	**(1)**	**(2)**
**NCTH SOFIA**	**41 858**	32.30	**RCTH PLOVDIV**	**26 678**	**41.97**
**RCTH PLOVDIV**	**26 678**	41.97	**RCTH PLEVEN**	8729	**40.54**
**RCTH VARNA**	13 687	31.28	**NCTH SOFIA**	**41 858**	32.30
DTH Burgas	9176	23.59	**RCTH VARNA**	13 687	31.28
**RCTH PLEVEN**	8729	40.54	DTH Smolyan	2600	28.72
**RCTH STARA ZAGORA**	7702	26.67	**RCTH STARA ZAGORA**	7702	26.67
DTH Blagoevgrad	7475	26.11	DTH Ruse	4914	26.41
DTH Pazardzhik	5353	23.95	DTH Blagoevgrad	7475	26.11
DTH Ruse	4914	26.41	DTH Lom/Montana	2790	24.75
DTH Sofia District	4248	18.91	DTH Yambol	2560	24.28
DTH Sliven	3875	22.89	DTH Pazardzhik	5353	23.95
DTH Haskovo	3588	17.30	DTH Burgas	9176	23.59
DTH Veliko Tarnovo	3362	16.78	DTH Sliven	3875	22.89
DTH Shumen	3152	21.11	DTH Silistra	1977	21.13
DTH Lom/Montana	2790	24.75	DTH Shumen	3152	21.11
DTH Kardzhali	2746	18.37	DTH Targovishte	1963	20.72
DTH Smoyan	2600	28.72	DTH Razgrad	1888	18.98
DTH Yambol	2560	24.28	DTH Sofia District	4248	18.91
DTH Dobrich	2523	17.38	DTH Kardzhali	2746	18.37
DTH Vratsa	2055	14.08	DTH Gabrovo	1723	18.37
DTH Silistra	1977	21.13	DTH Vidin	1262	17.89
DTH Targovishte	1963	20.72	DTH Dobrich	2523	17.38
DTH Razgrad	1888	18.98	DTH Haskovo	3588	17.30
DTH Pernik	1777	16.17	DTH Veliko Tarnovo	3362	16.78
DTH Gabrovo	1723	18.37	DTH Pernik	1777	16.17
DTH Kyustendil	1587	14.95	DTH Kyustendil	1587	14.95
DTH Lovech	1322	11.93	DTH Vratsa	2055	14.08
DTH Vidin	1262	17.89	DTH Lovech	1322	11.93

Again, in terms of basic descriptive statistics, the district-level data of blood donations per 1000 inhabitants exhibit a median of 21.125, a mean of 22.7789, a standard deviation of 7.2638 and a Gini coefficient of 0.1684, reflecting moderate variability without extreme outliers. Sofia, which in absolute terms was a significant outlier, in per-capita terms is furthest from the rest, but not a significant outlier (*p* > 0.05).

This means that once normalized, DTHs are much more similar in their donation activity than initially apparent. The pronounced inequalities observed in the total number of donations across Bulgarian districts are driven primarily by differences in district population size rather than by true variation in donor engagement. This engagement in per-capita numbers is visualized on the choropleth map in figure 6.

**Figure 6.**
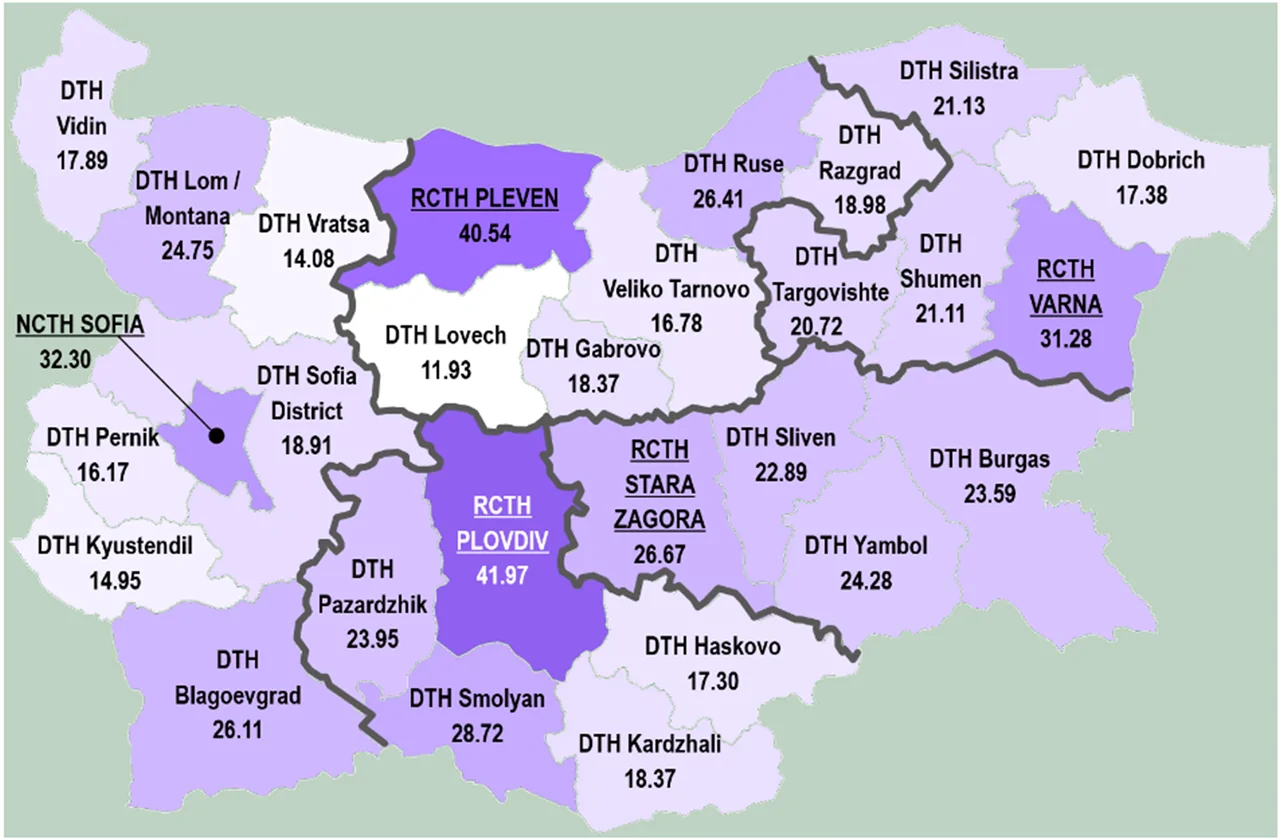
Choropleth map of Bulgaria regarding the number of blood donations per 1000 inhabitants.

### 3.3. Blood donations by donor's sex

Regarding blood donor's sex, it is noteworthy that in Bulgaria (as in many countries globally), there is a regulated limitation that men are permitted to donate blood no more than five times within a calendar year, and women no more than four times, with a minimum interval of two months between two donations [24]. Medical reasons for deferral include certain chronic diseases, recent health issues and vaccinations, chronic or recent use of certain medications, underweight, high-risk lifestyle factors, recent travels and procedures like tattoos and piercings [24]. Men are less likely to be temporarily deferred from donation on medical grounds, such as low haemoglobin levels or underweight, or to experience adverse reactions that could discourage future donation [25].

When the blood donor's sex is considered, patterns in European countries vary in wide margins: according to literature data, women represent 30% of donors in Italy, 33% in Greece, 43% in Portugal, 46% in Spain and Belgium, 50% in the Netherlands, Denmark and France and 55% in Finland [26].

In Bulgaria in 2024, women account for 24.85% of donors and men account for 75.15%, meaning that there is about one female donor for every three male donors. Despite the minor differences in the precise numbers, this one-to-three ratio is also present in the 2023 data [15]. District-wise, the highest proportions of female donors were registered in DTH Razgrad (30.72%) and RCTH Stara Zagora (30.17%) followed by DTH Lom (28.12%) and DTH Smolyan (28.00%), whereas the highest proportions of male blood donors were found in DTH Kardzhali (85.00%), DTH Haskovo (81.55%), DTH Vidin (81.54%) and DTH Lovech (79.76%). The complete data of blood donations by sex are provided in table 4 in the next section together with the data by type of donation.

**Table 4.** Blood donations by sex and type of donation, for all CTHs and DTHs in Bulgaria in 2024, in % (M—males, F—females, R—family/replacement and V—voluntary).

CTH/DTH of transfusion haematology	Total blood don.'s	Donations by sex		Donations by donor's sex and type of donation				Donations by type	
		M	F	M R	F R	M V	F V	R	V
**NCTH SOFIA**	**41 858**	74.97	25.03	62.28	20.42	10.64	4.00	82.71	14.64
DTH Blagoevgrad	7475	76.08	23.92	72.82	22.93	3.25	0.99	95.75	4.24
DTH Vidin	1262	81.54	18.46	80.59	18.38	0.95	0.08	98.97	1.03
DTH Vratsa	2055	77.71	22.29	75.18	21.46	2.53	0.83	96.64	3.36
DTH Kyustendil	1587	76.37	23.63	19.47	5.29	56.90	18.34	24.76	75.24
DTH Lom	1810	71.88	28.12	70.50	27.73	1.38	0.39	98.23	1.77
DTH Montana	980	76.22	23.78	75.92	23.47	0.31	0.31	99.39	0.61
DTH Pernik	1777	74.34	25.66	71.81	24.99	2.53	0.68	96.79	3.21
DTH Sofia District	4248	75.82	24.18	69.87	22.65	5.89	1.53	92.51	7.42
**RCTH PLOVDIV**	26 678	72.68	27.32	57.83	19.01	14.47	8.03	76.85	22.51
DTH Kardzhali	2746	85.00	15.00	80.52	14.17	4.48	0.84	94.68	5.32
DTH Pazardzhik	5353	79.49	20.51	76.26	19.24	3.01	1.20	95.50	4.20
DTH Smolyan	2600	72.00	28.00	64.85	25.65	7.15	2.35	90.50	9.50
DTH Haskovo	3588	81.55	18.45	77.70	17.50	3.85	0.92	95.21	4.77
**RCTH S. ZAGORA**	13 687	73.29	26.71	51.61	18.14	17.93	11.66	71.51	27.05
DTH Burgas	2523	78.52	21.48	72.46	18.33	6.71	1.82	97.50	2.50
DTH Sliven	1977	77.39	22.61	70.53	23.64	3.02	1.06	98.38	1.47
DTH Yambol	1963	74.53	25.47	71.13	22.42	4.73	1.05	93.63	5.20
**RCTH VARNA**	3152	74.18	25.82	53.58	17.93	18.51	8.53	82.52	16.24
DTH Dobrich	7702	69.83	30.17	76.26	21.24	2.26	0.24	69.75	29.59
DTH Silistra	9176	79.76	20.24	76.07	22.31	1.16	0.30	90.79	8.53
DTH Targovishte	3875	74.79	25.21	69.64	23.99	4.02	1.17	94.17	4.08
DTH Shumen	2560	76.33	23.67	61.07	21.45	12.15	4.09	93.55	5.78
**RCTH PLEVEN**	8729	73.26	26.74	68.21	24.56	5.05	2.17	92.77	7.22
DTH Veliko Tarnovo	3362	75.82	24.18	63.47	19.39	12.34	4.79	82.87	17.13
DTH Gabrovo	1723	77.31	22.69	73.07	22.29	4.12	0.35	95.36	4.47
DTH Lovech	1322	79.43	20.57	1.29	0.23	78.14	20.35	1.51	98.49
DTH Razgrad	1888	69.28	30.72	67.11	30.24	2.17	0.48	97.35	2.65
DTH Ruse	4914	79.18	20.82	76.54	19.88	2.63	0.94	96.42	3.56
**Total/Average**	**172 570**	**75.15**	**24.85**	**64.01**	**20.18**	**10.37**	**4.41**	**14.78**	**84.19**
								13.56*	85.39*

* Average values in the ‘donations by type’ columns are calculated in two different ways (see the text).

Regarding the findings from the statistics of blood donations by sex, it has been discovered (and reaffirmed with the authors’ previous study [15]) that Bulgaria demonstrates extremely high dependence on male blood donors. In addition to the legally imposed limitation of up to four donations per year for women, compared with up to five donations per year for men, this pattern may suggest possible health-screening barriers for women (anaemia, pregnancy and underweight), and some culturally gendered norms around donation in certain regions. Yet, it opens opportunities for building recruitment strategies specifically targeting female populations.

### 3.4. Blood donations by type of donation

In Bulgaria, two principal forms of blood donation are practised: voluntary non-remunerated donation and family/replacement donation. While the latter is also non-remunerated, it is directed towards a specific patient, typically at the request of the hospital, as a condition for providing treatment. Family/replacement donation thus constitutes a widely used mechanism to secure sufficient blood supplies in a healthcare system where the proportion of individuals donating voluntarily remains very low. Remunerated blood donation is also an eligible option, but one so rarely practised that these records have been safely disregarded from the presented statistical analysis.

A system based on voluntary, non-remunerated donation of blood and plasma is generally considered safer, as the incidence and prevalence of transfusion-transmissible infections are consistently lower than those observed among family or replacement donors and among paid donors [27,28]. Such systems also facilitate the implementation of donor education and selection measures that encourage individuals who may be unsuitable to defer or exclude themselves from donation [27].

For years, the WHO has been advocating globally for reduction in targeted and paid donations, and transition to 100% voluntary, non-remunerated donation as the most sustainable and safest model for blood supply [29]. Achieving this, however, requires not only a shift in attitudes and mindset towards voluntary donation—and volunteerism more broadly—but also specific legislative measures. Family/replacement donation remains predominant in countries with lower living standards, while in Western Europe, the USA and Canada, 70–90% of donations are fully voluntary and non-remunerated. In this respect, WHO reports have highlighted a concerning downward trend in voluntary donation in Bulgaria: 32.4% in 2004 [17, p. 43], 22.05% in 2007 [18, p. 9], 18.56% in 2011 [20, p. 71] and 24.01% in 2012 [20, p. 71].

Table 4 presents data on donor sex and type of donation for all districts in Bulgaria. According to the data from the NCTH information system for blood donation for 2024, summarized on a national scale, voluntary blood donation accounts for 14.78%, while the family/replacement type accounts for 84.19% of all blood donations, with a complementary 1.03% of special cases of blood donors, whose type of donation is apheresis or remunerated (this percentage is specifically higher in NCTH Sofia).

Notably, in two DTHs (Kyustendil and Lovech), the data differ significantly from all other 27 CTHs/DTHs. This pattern was also observed in the 2023 data [30] and is considered [15] to result from the incorrect input of primary data by the DTH staff, rather than a regional specificity such as a higher culture of donation or predominantly altruistic attitudes. Among the possible explanations for this recurrent misentry of data in these two regions are: insufficient training of the data operators and/or local misunderstanding of the terminology in use. In particular, this could be the effect of attributing blood donations as ‘family/replacement’ exclusively in cases where a direct family relationship between the donor and the intended recipient was inferred (e.g. on the basis of matching surnames).

Hence, if the data are summarized on a national scale *without* considering these two outlier DTHs, the volumes of voluntary and family/replacement types of blood donation would be re-estimated to 13.56% and 85.39%, respectively, with the complementary 1.05% of apheresis or remunerated donors. The data in table 4 show that, regardless of the method used to calculate the average estimate, 21 DTHs (out of 29 centres and departments) exhibit voluntary donation rates below this value. Above the estimate are the five CTHs, the two anomalous DTHs discussed above and DTH Dobrich, with a voluntary blood donation rate of 29.59%.

Another massively dominant pattern is that of family/replacement donation in most districts (national average, approx. 85% replacement versus 15% voluntary). Disregarding the two outlier DTHs with anomalously recorded data for the type of donation (Kyustendil and Lovech, as discussed above), the highest ratios of voluntary to replacement donation are identified traditionally in some of the largest cities of Bulgaria (Stara Zagora, 29.59%; Varna, 27.05%; Plovdiv, 22.51%; Veliko Tarnovo, 17.13%; Shumen, 16.24%; and Sofia, 14.64%). At the other extreme are DTHs like Vidin, Montana, Dobrich, Razgrad and Lom that rely almost exclusively on replacement donors—a clear signal of system vulnerability and absence of a sustainable voluntary donor culture. Part of the explanation is certainly in the fact that all of these largest cities are university towns with younger populations; some of the cities (Sofia, Plovdiv, Varna and Stara Zagora) have medical universities (which brings better blood donation awareness), as well as large multi-profile hospitals for active treatment, better equipped and human resourced to treat patients from the less populated adjacent districts. The latter results in cross-regional blood donors’ migration for family/replacement purposes.

Additionally, in some districts, it is noteworthy that although female blood donation is expectedly less than that of male, females are more prone to voluntary (non-targeted) blood donations. Averaged on a national level, voluntary donation in females is 17.76% compared with 13.80% voluntary donation in males. Respectively, the percentages of female voluntary donation are more pronounced in all CTHs (i.e. NCTH Sofia (15.99% versus 14.19%), RCTH Plovdiv (29.40% versus 19.91%), RCTH Varna (31.95% versus 25.26%), RCTH Stara Zagora (31.95% versus 25.26%) and RCTH Pleven (8.10% versus 6.90%)) and in three DTHs (Veliko Tarnovo (19.80% versus 16.28%), Pazardzhik (5.83% versus 3.78%) and Ruse (4.50% versus 3.32%)). On the other hand, the districts where voluntary blood donation by male donors prevails are Gabrovo (5.33% male versus 1.53% female voluntary blood donors), Razgrad (3.13% versus 1.55%), Smolyan (9.94% versus 8.38%), Sofia District (7.76% versus 6.33%), Targovishte (5.40% versus 4.60%), Pernik (3.41% vs 2.63%), Dobrich (2.88% versus 1.11%), etc. These figures can be indicative for the regions and districts where more focused strategies for targeting and recruitment of female donors need to be formulated.

### 3.5. Blood donations by donor's age

In Bulgaria, blood donation is permitted for healthy individuals between the ages of 18 and 65, a range that reflects both medical safety considerations and international standards for donor eligibility. This age interval is designed to ensure that donors possess sufficient physiological reserve to tolerate blood loss without adverse consequences, while minimizing risks associated with age-related health conditions that become more common after 65.

The lower limit of 18 aligns with legal capacity and the completion of adolescent physiological development. Within this range, donation patterns typically show that younger adults—especially those in their 20s and early 30s—tend to be less engaged, often owing to limited awareness, unstable routines or the absence of personal exposure to situations requiring blood transfusion. Many people in this age group do not yet have seriously ill elderly people in their inner circle who are in need of blood, which reduces the emotional or motivational triggers that often initiate first-time donation among older adults.

Conversely, middle-aged adults, particularly repeat donors, form the backbone of the donation system, providing a stable and reliable supply. This observation is in line with a 2019 study [31] from the Netherlands that showed that the highest donation willingness is found among Millennials (Generation Y) and Generation X, while discussing the design of targeted recruitment and retention strategies for improving evidence‐based donor management.

Bulgaria, like many European countries, faces the challenge of an ageing population: without continuous recruitment of younger donors, the sustainability of the donor pool may be threatened as large cohorts of people with rich blood donation practice approach the upper age limit. Thus, understanding and actively addressing age-related dynamics is essential for maintaining a resilient and self-sufficient blood donation system.

In what follows, the data and findings regarding patterns associated with the demographic indicator of age are presented. The analysis shows that in all CTHs and DTHs, the average age of all blood donors in 2024 varies between 37.4 and 45.5 years, with a country average of 39.6. The average age of male blood donors by district varies between 37.4 and 42.4 years with a country average of 39.0, while the average age of women by district varies between 39.0 and 45.5 years with a country average of 41.5. The average age of blood donors by district, differentiated by sex and taken altogether, is visualized in figure 7, while the age distribution of blood donations for the whole country by years (in the interval 18–65 years) is presented in figure 8.

**Figure 7.**
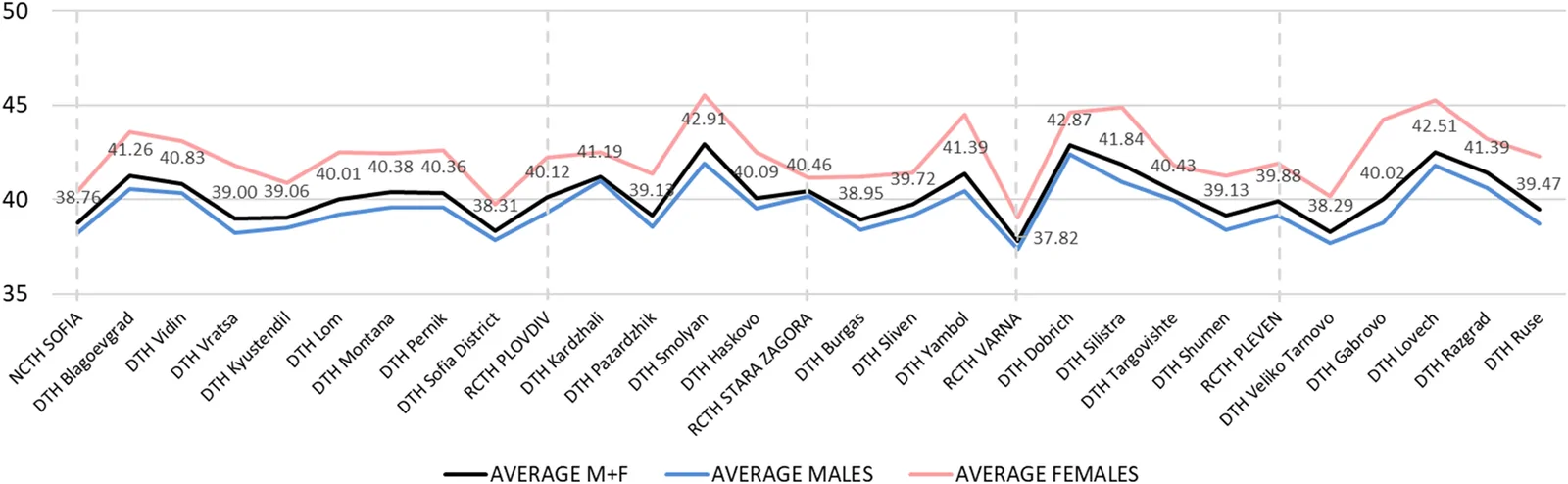
Average age of blood donors by sex by CTHs and DTHs in Bulgaria, 2024.

**Figure 8.**
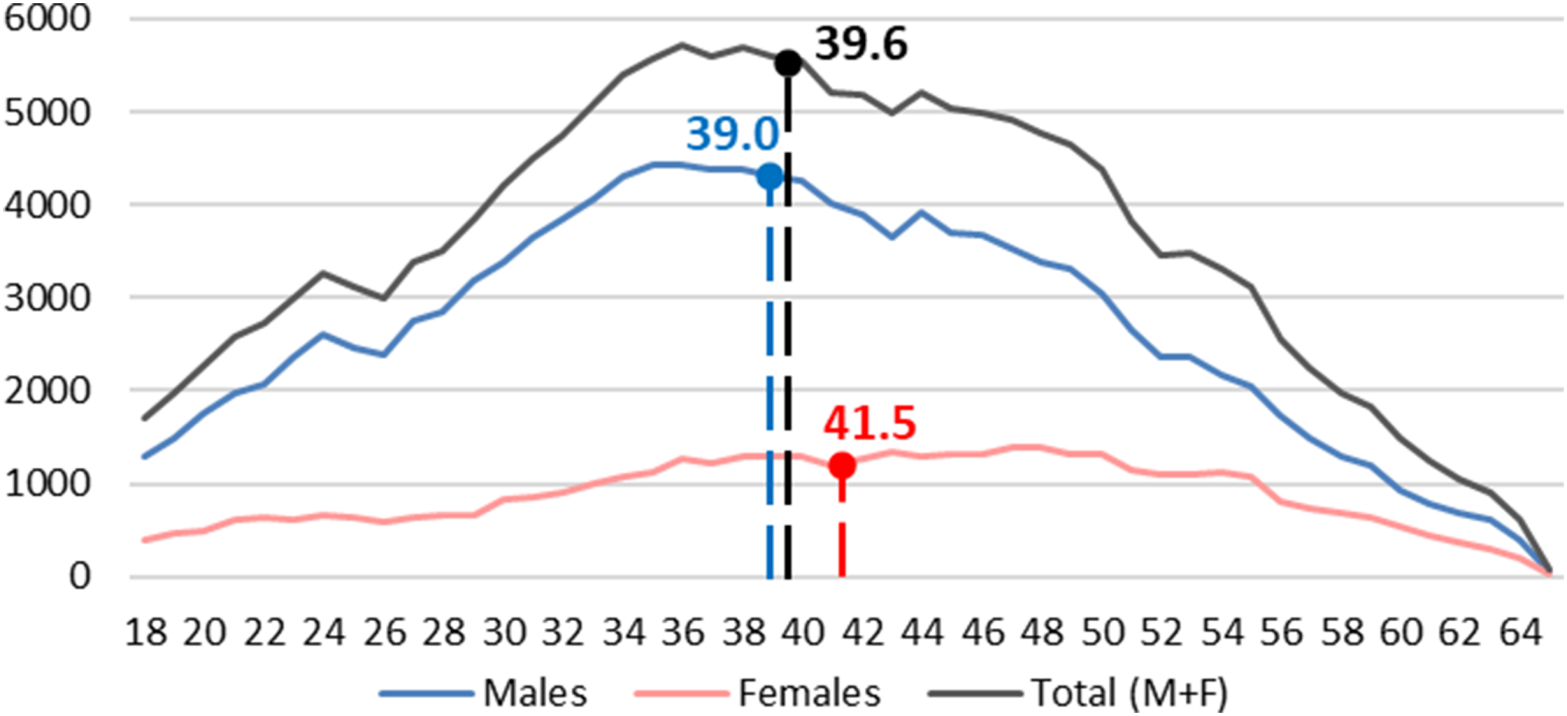
Average age of blood donors in Bulgaria by sex, 2024.

The graph in figure 8 clearly shows that both statistical distributions by age among male and female blood donors are most consistent with a low-variance normal (Gaussian-like) distribution.

The youngest blood donors are in DTH Varna (37.8 years average for both sexes, 37.4 years for males and 39.1 years for females) and DTH Veliko Tarnovo (38.3 /37.7/40.2), while the oldest are in DTH Smolyan (42.9/41.9/45.5), DTH Dobrich (42.9/42.4/44.6) and DTH Lovech (42.5/41.8/45.3). In 2023, the same DTHs featured in the same positions, despite having slightly different numbers [18].

Another way to approach the blood donation demographics of Bulgaria is to study the relations between donor's age and location. Figure 9 presents a heatmap visualization of the numbers of donations according to these two parameters, where the columns (CTHs/DTHs) are re-sorted in descending order by the total number of registered donations for the whole year 2024 (thus following a different logic from the one in which CTHs/DTHs were listed along the x-axis of figure 7), and the rows contain, respectively, the ages from the interval from 18 to 65, defining blood donation eligibility. Such an analysis of blood donors by age and district has not previously been conducted for the Bulgarian population; therefore, the data are not directly comparable with earlier studies. A review of the current literature suggests that this type of data visualization has not previously been used in this context and hence represents a novel contribution of the present study.

**Figure 9.**
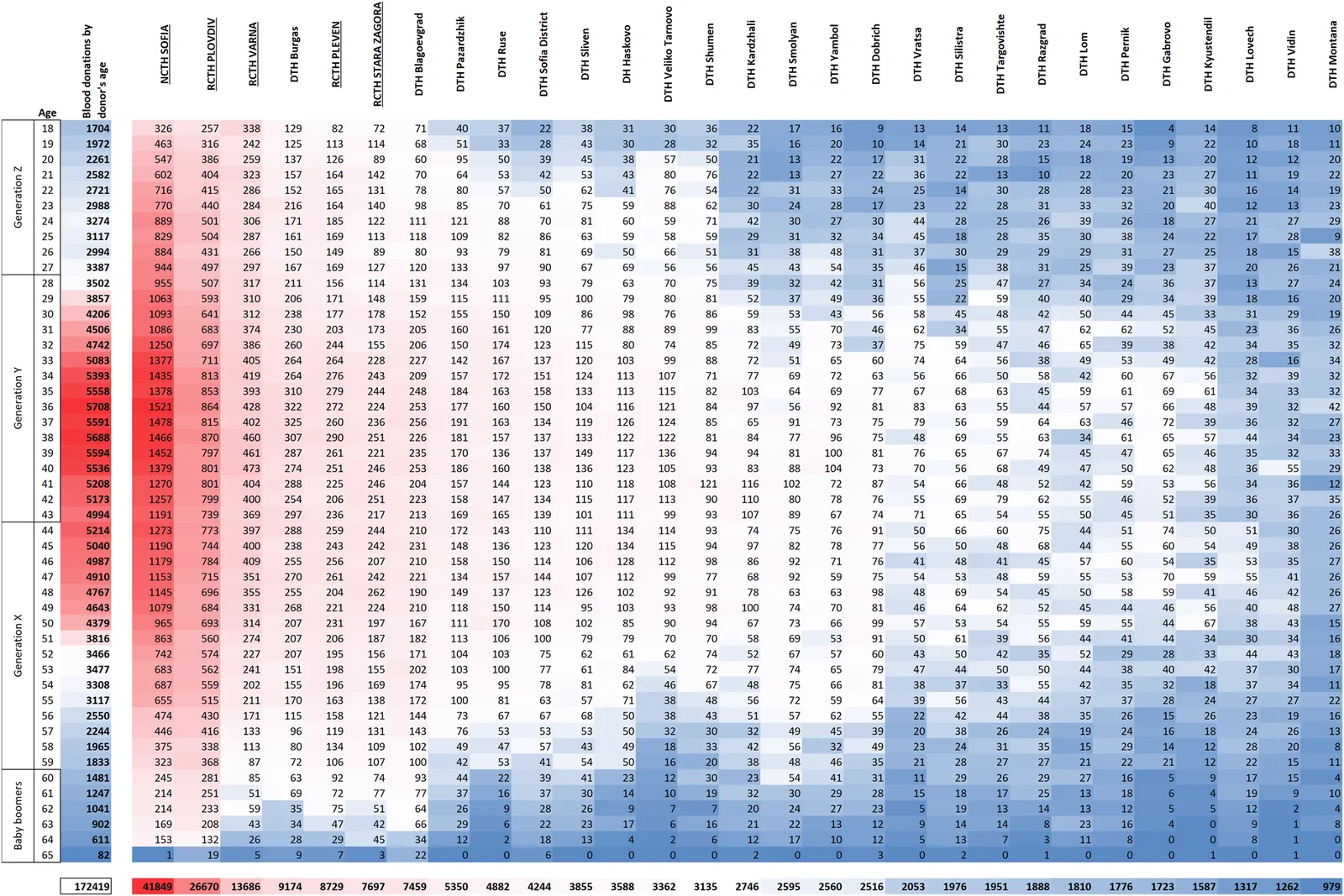
Blood donations by donor's age and by CTHs/DTHs in Bulgaria, 2024 (sorted in descending order by the total number of registered blood donations in districts).

The heatmap additionally includes the demographic cohorts (popularly termed as Baby Boomers, and Generations X, Y and Z, comprising the eligible donors aged 18 to 65).^2^ Conditional formatting applied to the data highlights larger values in shades of red and smaller values in shades of blue, but more importantly, it helps to outline unusual changes in behaviour or emerging trends. This donors’ age-based data analysis can be specifically useful when it serves as the basis for potential region- and age-related strategies for donor recruitment and retention. The following section outlines key considerations in the development of such strategies and targeted communication messages.

#### 3.5.1. Generation Z

Particular attention is given to strategies aimed at the youngest potential donors from the so-called Generation Z, who are approximately the 18–27-year-olds (typically defined as individuals born between 1997 and 2012, or from the mid-to-late 1990s until the early 2010s). Despite meeting eligibility criteria and being generally fit with respect to age and health, this group remains a relatively inactive segment of the blood donor population. Notably, in half of the DTHs in Bulgaria (from Kyustendil to Montana), Generation Z donors demonstrate markedly lower engagement compared with the rest of the country. Yet, young donors’ involvement should become an integral part of the local volunteering culture and practice, as these donors are expected to become the core of the next generation blood supply in the country. Since many Gen Z's are still students, a natural idea for consideration by relevant policymakers is organizing regular raising-awareness campaigns in high school and university settings, as part of the education journey. Being the first real ‘digital natives’, the representatives of this age group are strongly influenced by lead-by-example approaches to voluntary blood donation, including campaigns involving influencers and the presence of donation-related content on social media platforms [32,33], including TikTok [34]. Furthermore, as the first generation described as ‘mobile natives’, they are particularly responsive to mobile blood donation applications, especially those incorporating elements of gamification, and a substantial body of research in this area is already available [35–40].

#### 3.5.2. Generation Y

The second demographic cohort, that of Generation Y (also Millenials), comprises tentatively the donors aged 28 to 43 (or the generation defined as those born from the early 1980s until the mid-1990s to early 2000s) providing the largest share of donations and consequently determining the average ages of blood donors in Bulgaria for both sexes (figures 7 and 8). The heatmap from figure 9 very clearly outlines the sharp differences in donor engagement between Generation Y and Generation Z (see the aggregated data in the column ‘Blood donations by donor's age’).

When thinking about blood donors from this demographic cohort, it helps to consider both their life stage and motivations, specific digital habits, desire for a meaningful impact, need for trust and transparency and personal health awareness. Many people in this age range are typically working full-time and raising children, while balancing multiple responsibilities. This makes convenience a factor of crucial importance for this age cohort, so long waits or distant donation sites may significantly reduce their participation. Hence, an appropriate strategy for this target group is the mobile blood donation campaigns at workplaces or commercial centres, with specific consideration of the event's timing in either case. Workplace donations reduce time barriers and add a social element, which increases participation and influences both first-time donation and long-term retention, and that is critical for maintaining a stable and sustainable blood supply.

Additionally, it is noteworthy that Bulgarian Labour Code (Art. 157(1)(2)) grants two additional days off work (the day of the examination and donation, as well as the day thereafter) *independent of the employer*, a particularly ‘generous’ policy that is typical for Eastern Europe and Russia [41]. This paid leave not only helps by reducing time-related barriers, it is also consistent with incentives that matter to the representatives of Generation Y, who tend to value policies that acknowledge their contribution and social impact. Hence, a strategy oriented towards this age group should additionally raise public awareness of this measure, even though it is discussible whether it is aligned with the completely altruistic nature of voluntary blood donation. A recent study [42] conducted by some of the authors, examining temporal patterns of blood donation at RCTH Pleven during 2023–2024, found that the weekly peak of family/replacement donations occurs on Monday, whereas voluntary donations most frequently peak on Thursday, likely reflecting donors’ preference for ‘longer weekends’. Variations associated with bank holidays further supported this pattern.

In this regard, it is also worth considering the conclusions from a 2010 study [43] of the (then young) Gen Y's attitudes towards blood donation, where it was observed that the members of this generation are both altruistically motivated and opportunistic, ‘ready for volunteerism’, ‘as long as it is connected with [them] feeling good’. In other words, while Generation Y is open to blood donation, they are equally likely to engage in other charitable activities, thus making it important to highlight—and not compromise—the altruistic impact of donating.

#### 3.5.3. Generation X

The third demographic cohort, that of Generation X, comprises tentatively the donors aged 44 to 59 (or the generation of people born from the mid-1960s until the early 1980s). As can be seen from figure 9, people in this age group are still actively donating compared with the representatives from the other age groups, but this activity normally declines with age, as people get more prone to health-related issues and, respectively, deferrals.

Representatives of Generation X donors are considered to be often motivated by a sense of civic duty and social responsibility, as well as by maintaining their established donation habits. A typical trait of Gen X-ers, as discussed in [43], is their attitude of risk avoidance and low capacity for risks, combined with somewhat higher levels of distrust and scepticism. This being said, a prospective future research in this direction would be to investigate the rates of multi-time donors versus first timers in this age group.

On the other hand, this observation suggests that the important accents in the communication strategies targeting Generation X donors should be related to the blood centre's adherence to safety standards, convenient and predictable organization of blood donation, as well as the donor's personal regular health screening. While providing donors with the basic screening results of the collected blood is not a substitute for a full medical check-up and is only a secondary incentive for blood donation, it can be promoted as an additional motivating factor, owing to the perceived personal benefit and health awareness.

#### 3.5.4. Baby boomers generation

Finally, the generation of ‘Baby Boomers’ comprises tentatively the people born from 1946 to 1964, or in 2024, these were the donors aged 60 to 65. As figure 9 clearly shows, only in the five CTHs and two DTHs, there are more substantial shares of blood donors in this age cohort. Here, it is worth noting that the upper age limit of 65 in Bulgaria is not universal, and there are many countries that have raised or removed this limit to and over 70 [16]. In countries where healthy life expectancy is high, healthy repeat donors may be accepted at the discretion of the responsible physician (see [44] and references therein). At the same time, this may partly be a reflection of demographic changes: ageing populations increase clinical pressure and demand for blood while simultaneously reducing the proportion of younger donors, making the retention of healthy, experienced older donors an important strategy for maintaining blood supply.

As Baby Boomers and Generation X are more likely to have chronic illnesses in the family or to have undergone surgery or transfusion by themselves, this personal connection to the need for blood makes donation appeals more effective when they emphasize helping real patients or supporting the donor's local transfusion department and hospital. This aligns with a finding in [31] that knowing a transfusion recipient predicts and increases blood and plasma donation willingness across all age groups.

Finally, a factor that may discourage voluntary blood donation among potential voluntary donors of all age groups is the concern that a relative might require transfusion in the near future, while the donor would be temporarily ineligible to donate again because of the mandatory interval between two donations. This perception may often lead individuals to postpone or avoid voluntary donation in order to ‘reserve’ their ability to donate for a family member if needed. Such a misconception may be adequately addressed through wider dissemination of the otherwise existing policy that voluntarily donated blood can be *post factum* directed for potential use by relatives within a limited time frame after the donation act: usually one to six months in different CTHs of Bulgaria. Hence, a communication strategy based on more actively promoting this provision can enhance donor motivation by providing a perceived reciprocal family-directed benefit while maintaining the voluntary nature of donation.

In conclusion, it would be valuable to investigate which strategies for donor recruitment and retention are currently maintained or considered for implementation by local centres and DTHs in Bulgaria, the extent to which these strategies are aligned with the demographic profiles of actual and potential donors and how their effectiveness is assessed. The analysis presented here, together with the data-supported suggestions, may provide decision makers with a useful resource for informed future planning and may serve as a basis for further research and discussion on improving donor recruitment and retention policies in the country.

## 4. Conclusion

The present study analysed blood group distributions and indicators of blood donation activity in Bulgaria using a detailed, individual-level approach to data analysis. The results provide up-to-date evidence on the frequencies of ABO blood types, including the Rh(D) antigen, and on patterns of donation activity in relation to demographic characteristics, such as donor location, sex, age and type of donation (voluntary or family/replacement), considered both separately and in combination.

With respect to blood group distributions according to the ABO system and the Rh(D) antigen, the average values for the country are O(–) 4.47%, O(+) 29.37%, A(–) 5.21%, A(+) 36.35%, B(–) 2.15%, B(+) 14.50%, AB(–) 1.00% and AB(+) 6.95%, and in aggregated form, these are O: 33.84%, A: 41.56%, B: 16.65% and AB: 7.95%. Over time, these frequencies appear relatively stable for blood groups B and AB, while group O shows a modest increase and group A shows a corresponding decrease compared with published data from the previous decade.

The study also provides a detailed overview of donors’ demographic characteristics and their preferences for voluntary or replacement donation. Based on the interpretation of the presented data, several considerations regarding appropriate recruitment and retention strategies were formulated in relation to donors’ age (generation), sex and geographic location. The individual-level analytical approach applied to data from the National Transfusion Haematology Centre of Bulgaria made it possible to identify patterns at district level and to obtain a more granular understanding of donation behaviours.

Some data-visualization methods that are not widely used in transfusion haematology literature were employed here to facilitate interpretation and to highlight patterns that may be less apparent in conventional presentation formats. Comparisons with previously published studies from Bulgaria place the findings in context and help to suggest possible explanations for the observed trends. Overall, the results contribute to immunohaematological research, to the study of voluntary blood donation activity and to the growing body of work demonstrating the value of analytics-driven approaches in health-system management. The evidence and conclusions presented here may support strategic planning related to the collection, allocation and logistics of blood and blood products, the organization of donation campaigns and the improvement of public awareness regarding the needs and benefits of voluntary blood donation in Bulgaria.

At the same time, the findings underline the continuing need for investment in education, infrastructure and targeted incentives aimed at encouraging regular voluntary donation. Strengthening the recruitment and retention of both donors and specialized personnel in transfusion haematology centres, together with the optimization of already limited resources, will remain essential for ensuring a stable and sustainable blood supply in the future.

## Supplementary Material



## Data Availability

A repository of data has been provided as electronic supplementary material [45].

## Footnotes

^1^

**Remark 1:** *The only notable exception from the principle that a District's administrative centre coincides with the centre/department of transfusion haematology is Montana District, where traditionally, until the year 2024, the single District's DTH used to be located in the town of Lom, while as of 2024, a new separate DTH was launched in the town of Montana, the District's administrative centre.*

^2^

**Remark 2:** *The total number of donations in these statistics is slightly smaller (172 419 versus 172 570) owing to a small number of foreign blood donors whose age is not explicitly recorded and are thus not included in this dataset.*

## Theme

One contribution of 9 to a theme issue ‘Data driven modelling for living systems’.
